# Diagnostic Capabilities of MRI and CT in Evaluating Dizziness: A Systematic Review of Acute Cases in the ED

**DOI:** 10.7759/cureus.88057

**Published:** 2025-07-16

**Authors:** Elsuha Elgassim Ali Mohammed, Mohamed Almogtaba Mohamed Alzain Ali, Ethar Eltayeb, Lobaba Mubarak Saidahmed Ahmed, Dalia Hamdan Ahmed Dafaalla, Mohammed Omer Mohammed Elsheikh, Hanady ME M Osman

**Affiliations:** 1 Radiology, Shendi University, Shendi, SDN; 2 General Practice, Thumbay Clinic, Sharjah, ARE; 3 Endocrinology and General Medicine, University Hospital Limerick, Limerick, IRL; 4 General Practice, Shendi University, Shendi, SDN; 5 Emergency Medicine, The Shrewsbury and Telford Hospital NHS Trust, Shrewsbury, GBR; 6 Orthopedics, Health Service Executive, Galway, IRL; 7 Quality and Patient Safety, Najran Armed Forces Hospital, Ministry of Defense Health Services, Najran, SAU

**Keywords:** ct, diagnostic accuracy, dizziness, emergency department, mri, posterior fossa, stroke, vertigo

## Abstract

Dizziness is a common reason for ED visits, posing diagnostic challenges due to its broad range of potential causes, from benign vestibular conditions to critical cerebrovascular events. Although CT scans are often used to quickly assess for intracranial hemorrhage, MRI provides greater accuracy for identifying strokes in the posterior circulation. Differences in imaging practices and uncertainty about the most effective approach highlight the need for a thorough evaluation of these modalities. This narrative systematic review examined the diagnostic performance of MRI and CT in assessing patients presenting with acute dizziness in ED settings, focusing on detection rates and clinical considerations. A comprehensive literature search was conducted, and eight relevant studies were included. The methodological quality of the studies was assessed, and findings were synthesized narratively due to variability in study designs. Overall, MRI showed a higher detection rate for underlying causes of dizziness compared to CT, particularly for posterior circulation strokes. CT was mainly useful for ruling out hemorrhage, while MRI offered superior detection of ischemic events. Using clinical factors such as age, vascular risk, and neurological findings may help prioritize MRI use in patients with higher stroke risk, supporting targeted imaging strategies to improve diagnostic outcomes and resource utilization.

## Introduction and background

Dizziness is among the most frequently reported symptoms in EDs worldwide, accounting for approximately 4-5% of all ED visits [1]. Clinically, dizziness is an umbrella term encompassing sensations such as vertigo (the false sense of movement), presyncope (feeling of impending faint), disequilibrium (imbalance), and nonspecific lightheadedness [2]. Although often perceived as benign, dizziness can signal a spectrum of underlying etiologies ranging from peripheral vestibular disorders, such as benign paroxysmal positional vertigo, to life-threatening central nervous system pathologies, including ischemic stroke or brain hemorrhage [2]. The broad differential diagnosis, combined with its nonspecific nature, poses a significant challenge to emergency physicians, particularly when making time-sensitive decisions under diagnostic uncertainty [3].

In routine clinical practice, bedside diagnostic tools such as the head impulse, nystagmus, test of skew (HINTS) examination, the STANDING protocol, and risk stratification scores like ABCD2 are used to differentiate peripheral from central causes of dizziness, especially in suspected stroke [4]. However, these examinations require specific expertise and may not always be conclusive, leading to frequent reliance on neuroimaging. Over the past two decades, MRI and CT have emerged as cornerstones in the diagnostic workup of acute dizziness [4]. While CT remains the most widely used modality in ED settings due to its accessibility, speed, and utility in ruling out hemorrhage, it is often limited in sensitivity for detecting posterior circulation infarcts [4,5]. Conversely, MRI, particularly with diffusion-weighted imaging (DWI), offers superior sensitivity for ischemic stroke, especially in the posterior fossa, but is often constrained by availability, longer acquisition times, cost, and contraindications in certain patient populations [2].

Current clinical practice is further complicated by variability in imaging protocols, inconsistent use of decision rules, and a lack of consensus regarding the optimal imaging strategy for dizzy patients in the ED [6]. The decision to use MRI or CT - and when to deploy each - remains largely clinician-dependent and influenced by institutional resources [7]. Moreover, while both imaging modalities have been extensively studied, there remains a paucity of synthesized evidence focusing specifically on their diagnostic performance in acute dizziness cases within emergency settings [8].

In light of these diagnostic complexities and the imperative to rapidly and accurately differentiate benign from serious causes of dizziness, it is essential to assess the current landscape of imaging utilization [9]. A systematic evaluation of the diagnostic capabilities - namely, sensitivity, specificity, predictive values, and accuracy - of MRI and CT in this context can help guide evidence-based imaging strategies and improve patient outcomes.

The aim of this systematic review is to critically assess and compare the diagnostic performance of MRI and CT for evaluating dizziness in acute cases presenting to EDs, with a focus on reported diagnostic metrics, clinical relevance, and implications for practice.

## Review

Methodology

Protocol and Registration

This systematic review was conducted in accordance with the Preferred Reporting Items for Systematic reviews and Meta-Analyses (PRISMA) 2020 guidelines [10]. The protocol for this systematic review was not registered on PROSPERO because the review was part of an internal academic requirement with constrained timelines.

Eligibility Criteria

To ensure the relevance and consistency of the included studies, strict eligibility criteria were applied. Studies were eligible if they assessed the diagnostic performance of MRI and/or CT in adult patients presenting with dizziness or vertigo in an ED setting. Both prospective and retrospective observational studies, cross-sectional studies, and diagnostic accuracy studies were considered for inclusion. Only studies that reported at least one diagnostic performance metric - such as sensitivity, specificity, positive predictive value (PPV), negative predictive value (NPV), or overall accuracy - were included. Studies focused exclusively on pediatric populations, non-acute or chronic dizziness, outpatient settings, or imaging modalities other than MRI or CT (such as PET or ultrasound) were excluded. Additionally, reviews, editorials, conference abstracts, and non-peer-reviewed reports were not considered. Only studies published in English were included in the final synthesis.

Information Sources

The literature search was performed across five major electronic databases: PubMed, British Medical Journals (BMJ database), Scopus, IEEE Xplore, and Web of Science. These databases were chosen to capture a comprehensive range of both clinical and technical studies related to imaging modalities and diagnostic evaluation. The final search was completed on May 10, 2025, and the reference lists of all included studies were manually reviewed to identify additional relevant publications.

Search Strategy

A systematic and comprehensive search strategy was developed using a combination of Medical Subject Headings (MeSH) and free-text terms. The search included terms such as “dizziness”, “vertigo”, “emergency department”, “MRI”, “CT”, “computed tomography”, “magnetic resonance imaging”, “diagnostic accuracy”, “sensitivity”, and “specificity”. Boolean operators (AND/OR) were used to combine terms appropriately. The search strategy was tailored for each database to optimize sensitivity and specificity. Detailed search strings for each database are provided in the appendices to ensure reproducibility.

Selection Process

The selection of studies involved two independent reviewers (LMSA and DHAD) from the list of authors who screened all retrieved titles and abstracts based on the predefined eligibility criteria. Full-text articles were obtained for studies that met the initial screening criteria or where relevance could not be determined from the abstract alone. Disagreements between reviewers were resolved through discussion, and a third reviewer (MOME) was consulted when consensus could not be reached. The selection process is documented in a PRISMA 2020 flow diagram provided in the results section.

Data Collection Process

A structured data extraction form was designed and pilot-tested by the review team. Two reviewers independently extracted data from the included studies. Extracted information included study title, first author, year of publication, country, study design, sample size, patient demographics, imaging modality (MRI or CT), indication for imaging, diagnostic criteria used, reference standard for final diagnosis, and diagnostic metrics such as sensitivity, specificity, PPV, NPV, and accuracy. Any discrepancies in data extraction were discussed and resolved by mutual agreement, with input from a third reviewer when necessary. If critical data were missing or unclear, study authors were contacted for clarification.

Risk of Bias Assessment

The methodological quality of each included study was evaluated using the Quality Assessment of Diagnostic Accuracy Studies-2 (QUADAS-2) tool [11], which is specifically designed to assess risk of bias in diagnostic accuracy studies. The tool evaluates four key domains: patient selection, conduct and interpretation of the index test, reference standard, and flow and timing. Each domain was assessed for risk of bias and applicability concerns, and a summary of these assessments is presented in tabular and graphical formats. Two reviewers (LMSA and DHAD) conducted the assessment independently, and disagreements were resolved by consensus.

Data Synthesis and Justification for Not Conducting Meta-Analysis

Due to considerable clinical and methodological heterogeneity among the included studies, a quantitative meta-analysis was deemed inappropriate. Variations in study design, patient populations, imaging protocols, diagnostic thresholds, and outcome measures limited the comparability of findings across studies. Pooling such heterogeneous data would compromise the validity and generalizability of the results. Therefore, a narrative synthesis was conducted instead. This approach allowed for a detailed qualitative comparison of the diagnostic capabilities of MRI and CT, highlighting trends, patterns, and gaps in the current literature.

Reporting Bias and Certainty of Evidence

Given the limited number of studies and the heterogeneity in reporting outcomes, a formal assessment of publication bias through funnel plots or statistical tests was not feasible. The overall certainty of evidence was discussed narratively for each key outcome, considering the quality and consistency of the included studies, the risk of bias assessments, and the applicability of the results to real-world ED settings.

Results

Studies Selection Process

**Figure 1:**
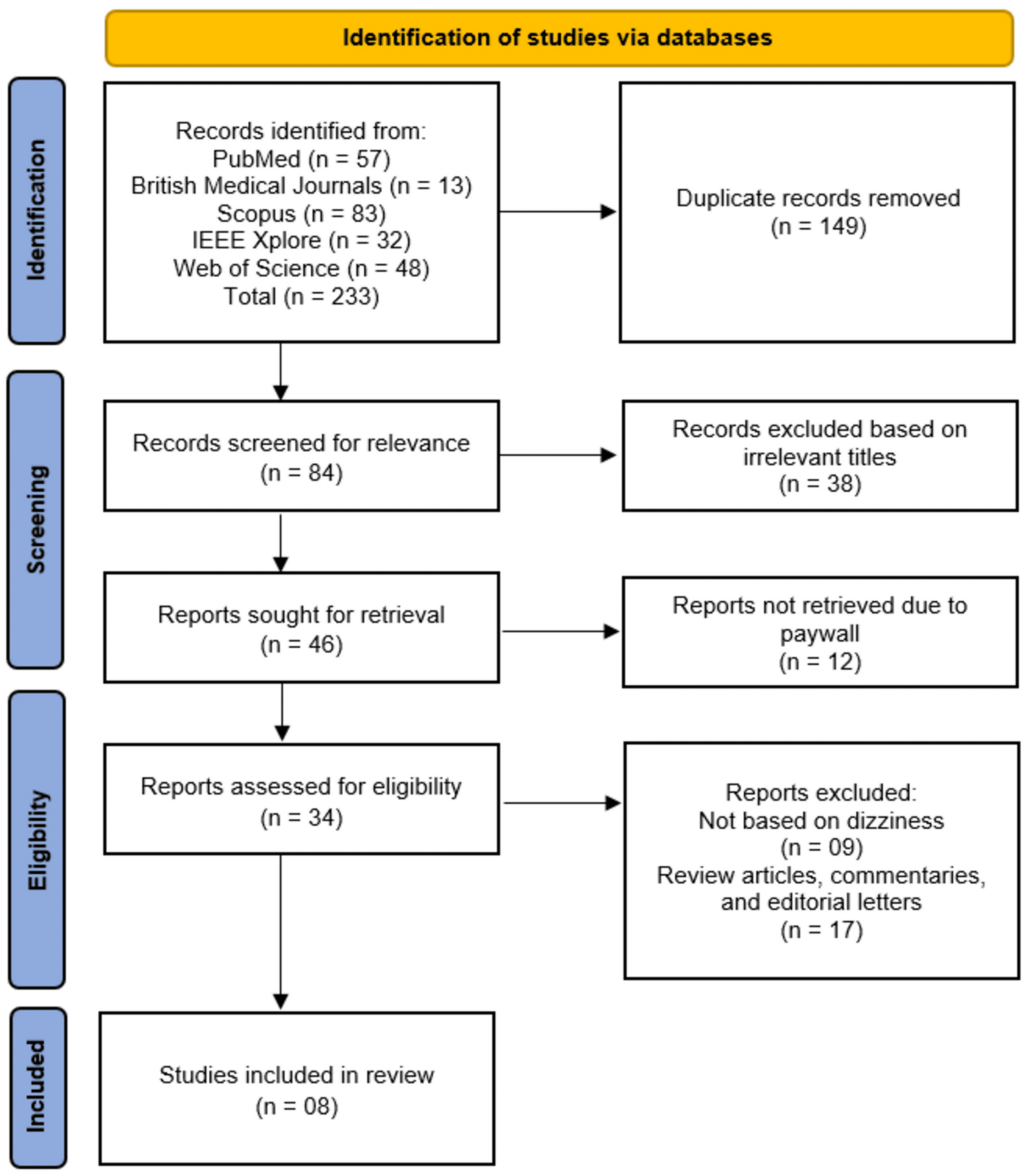
PRISMA diagram summarizing the literature search and study selection for included articles PRISMA, Preferred Reporting Items for Systematic reviews and Meta-Analyses

The initial literature search across five databases (PubMed, British Medical Journals, Scopus, IEEE Xplore, and Web of Science) yielded 233 records, from which 149 duplicates were removed. The remaining 84 records were screened for relevance, resulting in the exclusion of 38 studies based on irrelevant titles. Of the 46 reports sought for retrieval, 12 were unavailable due to paywall restrictions. The remaining 34 full-text articles were assessed for eligibility, with 26 excluded (nine for not focusing on dizziness and 17 for being review articles, commentaries, or editorial letters). Ultimately, eight studies [12-19] met the inclusion criteria and were incorporated into the systematic review. This rigorous selection process ensured that only the most relevant and methodologically sound studies were included (Figure 1).

Studies Characteristics

The systematic review included eight studies investigating the diagnostic capabilities of MRI and CT for dizziness in acute ED settings. The studies were conducted across diverse geographical locations, including Finland, Jordan, the USA, and Bahrain, and were published between 2012 and 2025. All studies employed retrospective designs, except for one prospective database analysis by Hwang et al. [17]. Sample sizes ranged from 67 to 1681 patients, with most studies focusing on patients presenting with dizziness, vertigo, or vague neurologic symptoms. The imaging modalities evaluated included MRI (emergency neuroimaging, thin axial/coronal DWI, and contrast-enhanced MRI), CT (non-contrast and CT angiography), and specialized techniques like MRI of internal auditory canals (MRIAC). Key indications for imaging were the detection of stroke, posterior fossa infarcts, or other acute pathologies (Table 1).

**Table 1:** Characteristics of the studies included in the systematic review assessing the diagnostic capabilities of MRI and CT AUC, area under the curve; CE-MR, contrast-enhanced magnetic resonance; CTA, CT angiography; DWI, diffusion-weighted imaging; FLAIR, fluid-attenuated inversion recovery; MRIAC, MRI of internal auditory canals; NCCT, non-contrast CT; SWI, susceptibility-weighted imaging; TIA, transient ischemic attack

First author and year	Country	Study design	Sample size	Patient population	Setting	Imaging modality (MRI/CT)	Indication for imaging	Key findings	Outcomes measured
Happonen et al. (2024) [12]	Finland	Retrospective study	1169	Patients with acute dizziness or vertigo	Tertiary hospital ED	MRI (emergency neuroimaging)	Detection of stroke or other acute pathology	17% had acute stroke; 8% other significant pathology; 75% normal findings; predictors for pathology: older age, male sex, cardiovascular risk factors, neurologic signs; isolated dizziness not discriminative	Positive imaging for acute stroke or other significant pathology; risk score performance (AUC for ischemic stroke 0.75, AUC for any pathology 0.70)
Alawneh et al. (2021) [13]	Jordan	Retrospective review	326	Patients presenting with dizziness or vertigo	ED of a tertiary university hospital	CT (all patients); MRI (follow-up for some)	Evaluation of dizziness/vertigo	CT detected abnormalities in 15% (mostly ischemic stroke); MRI had higher abnormal findings (27%); CT was less effective than MRI for dizziness evaluation	Detection of abnormal findings (ischemic stroke); correlation of lab and symptom data with vertigo
Hammoud et al. (2016) [14]	USA	Retrospective analysis	252	Patients presenting to the ED with vague neurologic symptoms (e.g., dizziness and altered mental status) and a negative head CT	ED	Head CT followed by head MRI	Atypical stroke symptoms with negative CT to detect minor infarcts	11.5% had acute/subacute infarct on MRI; positive MRI findings correlated with older age, hyperlipidemia, hypertension, diabetes, anticoagulation, and prior TIA/stroke	Positive MRI findings (acute/subacute infarct) and associated patient risk factors
Fakhran et al. (2013) [15]	USA	Retrospective	228 CTAs, 304 CE-MRs, 266 MRIACs (total: 798 studies)	Patients presenting with isolated dizziness; no signs of other neurologic pathology or known cause of dizziness	Emergency and outpatient settings	CTA of head and neck, contrast-enhanced brain MRI, contrast-enhanced MRIAC and temporal bones	Evaluation of isolated dizziness	Very low yield for significant findings (2.2% CTA, 1.3% CE-MR, 1.5% MRIAC); minimal impact on management (TE ~1.0%)	Proportion of abnormal findings, nature of abnormality, impact on clinical management
Ahsan et al. (2013) [16]	USA	Retrospective chart review	1681	Patients presenting with dizziness and vertigo with a specific health maintenance insurance plan	ED of a large health system	CT and MRI	Evaluation of dizziness and vertigo	CT scans had low predictive value for significant pathology (0.74% clinically significant); MRI detected significant abnormalities in 12.2% of cases; older patients more likely to receive CT	Usefulness of CT and MRI, diagnostic yield, costs, and predictors for imaging utilization
Hwang et al. (2012) [17]	USA	Prospective database analysis	67 patients	Acute ischemic stroke patients with a posterior fossa infarct	ED	NCCT and MRI	Suspected posterior fossa stroke within 30 h of symptom onset	NCCT had low sensitivity (41.8%) for detecting posterior fossa infarcts; MRI is more reliable in this context	Sensitivity of NCCT; time interval between symptom onset and detection
Masood et al. (2024) [18]	Bahrain	Retrospective review	481	Patients presenting to the ED with dizziness	ED at Salmaniya Medical Complex	CT	Assessment of acute dizziness	Majority (93.1%) had normal CT; 5.4% infarcts, 1.1% hemorrhages, 0.4% space-occupying lesions	Diagnostic yield of CT scans; prevalence of positive findings
Lozano et al.(2025) [19]	USA	Retrospective cohort study	615	Patients presenting to the ED with vertigo/dizziness	ED at a single institution	MRI (3 mm axial DWI, 3 mm coronal DWI, axial FLAIR, axial SWI)	Evaluation for ischemic stroke in patients with dizziness	Thin axial DWI had 100% sensitivity for detecting ischemia, whereas thin coronal DWI had lower sensitivity (83%); adding coronal DWI did not significantly improve detection	Sensitivity of thin axial vs. coronal DWI for ischemic lesion detection

Happonen et al. [12] and Alawneh et al. [13] highlighted the superiority of MRI over CT in detecting acute pathologies, with MRI identifying abnormalities in 17-27% of cases compared to CT’s lower yield (15% or less). Hammoud et al. [14] and Fakhran et al. [15] emphasized MRI’s role in identifying infarcts missed by CT, particularly in patients with atypical stroke symptoms. Meanwhile, Ahsan et al. [16] and Masood et al. [18] reported limited diagnostic value for CT in unremarkable clinical cases, with most scans (93.1%) being normal. Lozano et al. [19] demonstrated the high sensitivity of thin axial DWI (100%) for detecting ischemic lesions, outperforming coronal DWI (83%).

Diagnostic Performance of MRI and CT

MRI consistently demonstrated higher sensitivity and diagnostic yield compared to CT across studies. For instance, Hwang et al. [17] reported a sensitivity of only 41.8% for NCCT in detecting posterior fossa infarcts, whereas MRI was more reliable. Similarly, Lozano et al. [19] found that thin axial DWI achieved 100% sensitivity for ischemic stroke detection, underscoring MRI’s advantage in posterior circulation evaluation (Table 2).

**Table 2:** Summary of reported diagnostic performance metrics (sensitivity, specificity, predictive values, and accuracy) for MRI and CT AUC, area under the curve; CE-MR, contrast-enhanced magnetic resonance; CTA, CT angiography; DWI, diffusion-weighted imaging; IAC, internal acoustic canal; MRIAC, MRI of the internal acoustic canals; NCCT, non-contrast CT; NPV, negative predictive value; NR, not reported; PPV, positive predictive value; TIA, transient ischemic attack

Author and year	Imaging modality	Sensitivity (%)	Specificity (%)	PPV (%)	NPV (%)	Accuracy (%)	Key diagnostic findings
Happonen et al. (2024) [12]	MRI (emergency)/CT/internal acoustic canal MRI	Risk score AUC for stroke = 0.75, for any significant pathology = 0.70; overall moderate for MRI, low for CT, and canal MRI	Moderate for MRI, low for CT, and canal MRI	NR	NR	NR	MRI detected acute stroke in 17% and other pathology in 8% (total 25% positive); CT and canal MRI showed low diagnostic yield; predictors like older age, male sex, cardiovascular risk factors, and neurologic signs improve yield and may guide imaging selection.
Alawneh et al. (2021) [13]	CT: ~15% abnormal findings; MRI: ~27% abnormal findings	NR	NR	NR	NR	CT: low MRI: higher	CT detected abnormalities in 15% (mostly ischemic stroke); MRI detected abnormalities in 27% and was more effective for dizziness evaluation in the ED.
Hammoud et al. (2016) [14]	NR	NR	NR	NR	NR	NR	CT: negative in 252 patients with vague neurologic symptoms; missed 11.5% cases of acute/subacute infarct. MRI: detected infarcts in 11.5% (29/252) missed by CT; positive MRI associated with older age, hypertension, hyperlipidemia, diabetes, anticoagulation use, prior TIA/stroke.
Fakhran et al. (2013) [15]	CTA (head and neck), CE-MR (brain), MRIAC (IAC and temporal bones)	NR	NR	NR	NR	NR	CTA: 2.2% significant findings, 1.3% changed management; CE-MR: 1.3% significant findings, 0.7% changed management; MRIAC: 1.5% significant findings, 1.1% changed management; Overall therapeutic efficacy ~1%
Ahsan et al. (2013) [16]	NR	NR	NR	NR	NR	CT: low predictive value for significant pathology; only 0.74% showed findings requiring intervention; high cost, low yield. MRI: higher yield than CT (12.2% significant abnormalities); recommended for patients with additional neurological signs; not practical for all dizziness cases in the ED.	CT: very low yield for significant pathology (0.74%); high cost with little benefit; MRI: higher yield (12.2% significant findings); recommended when additional neurological signs are present; not cost-effective for routine dizziness in ED.
Hwang et al. (2012) [17]	NCCT	41.80%	NR	NR	NR	NR	Frequently insensitive for detecting posterior fossa infarction; sensitivity limited by the temporal evolution of stroke and beam-hardening artifact. MRI is preferred when NCCT is nondiagnostic.
Masood et al. (2024) [18]	CT	NR	NR	NR	NR	NR	Majority (93.1%) normal; 5.4% infarcts; 1.1% hemorrhages; 0.4% space-occupying lesions. Limited diagnostic value in patients with an unremarkable clinical exam.
Lozano et al.(2025) [19]	Thin axial DWI (3 mm) and thin coronal DWI (3 mm)	Thin axial DWI: 100% (95% CI: 95-100); Thin Coronal DWI: 83% (95% CI: 72-91)	NR	NR	NR	NR	Thin axial DWI detected all ischemic lesions in patients with dizziness in the ED; thin coronal DWI missed smaller lesions (2-8 mm); adding coronal DWI did not improve diagnostic yield; replacing conventional 5 mm DWI with thin axial DWI is recommended for better detection of posterior circulation ischemia.

CT’s limitations were evident in multiple studies. Ahsan et al. [16] noted a very low predictive value for significant pathology (0.74%), while Fakhran et al. [15] reported minimal therapeutic efficacy (≤2.2%) for CT angiography and contrast-enhanced MRI. In contrast, MRI detected clinically significant abnormalities in 12.2-27% of cases, particularly in older patients or those with cardiovascular risk factors [13,14].

Key Predictors and Clinical Implications

Several studies identified predictors to guide imaging selection. Older age, male sex, cardiovascular risk factors, and neurologic signs were associated with higher yields for MRI [12,14]. Isolated dizziness without these predictors often resulted in normal imaging findings, suggesting selective use of MRI in high-risk populations. Cost-effectiveness was another concern; Ahsan et al. [16] cautioned against routine MRI for dizziness due to high costs, recommending it only for patients with additional neurological signs.

Results of Risk of Bias Assessment

The risk of bias assessment, conducted using the QUADAS-2 tool, revealed variability across the included studies. Most studies demonstrated low risk in patient selection [12,13,17-19], index test performance (all studies), and flow and timing (all except Fakhran et al. [15], which had high risk due to delays between imaging modalities). However, concerns arose in the reference standard domain, where Hammoud et al. [14] had a high risk due to unblinded MRI interpretation, and Fakhran et al. [15] and Ahsan et al. [16] were rated unclear due to a lack of independent verification. Overall, five studies were deemed low risk [12,13,17-19], and two high risk [15,16]. These findings suggest that while MRI’s diagnostic superiority is supported by robust studies, conclusions from higher-risk studies should be interpreted with caution (Table 3).

**Table 3:** Quality assessment using the QUADAS-2 tool QUADAS-2, Quality Assessment of Diagnostic Accuracy Studies-2

Study and year	Patient selection	Index test (MRI/CT)	Reference standard	Flow and timing	Overall risk of bias
Happonen et al. (2024) [12]	Low	Low	Low	Low	Low
Alawneh et al. (2021) [13]	Low	Low	Low	Low	Low
Hammoud et al. (2016) [14]	Moderate	Low	High	Low	Moderate
Fakhran et al. (2013) [15]	High	Low	Unclear	High	High
Ahsan et al. (2013) [16]	High	Low	Unclear	Moderate	High
Hwang et al. (2012) [17]	Low	Low	Low	Low	Low
Masood et al. (2024) [18]	Low	Low	Low	Low	Low
Lozano et al. (2025) [19]	Low	Low	Low	Low	Low

Discussion

The findings of this systematic review underscore the superior diagnostic capabilities of MRI compared to CT in evaluating acute dizziness and vertigo in ED settings, particularly for detecting posterior fossa infarctions and other acute pathologies. Across the eight included studies, MRI consistently demonstrated higher sensitivity and diagnostic yield, with detection rates for significant abnormalities ranging from 12.2% to 27%, compared to CT’s lower yield of 0.74-15% [12,13,16]. This aligns with existing literature emphasizing MRI’s superiority in identifying ischemic strokes, especially in posterior circulation territories, where CT’s sensitivity is limited by beam-hardening artifacts and lower resolution [20,21]. For instance, Hwang et al. [17] reported a sensitivity of just 41.8% for NCCT in detecting posterior fossa infarcts, while MRI reliably identified these lesions, reinforcing its role as the gold standard for acute stroke evaluation. Similarly, Lozano et al. [19] demonstrated that thin axial DWI achieved 100% sensitivity for ischemic lesions, far outperforming coronal DWI (83%), which suggests that optimized MRI protocols can further enhance diagnostic accuracy in dizziness evaluation.

The limited diagnostic utility of CT in this context is a recurring theme, particularly for patients with isolated dizziness and no focal neurologic deficits. Studies such as those by Masood et al. [18] and Ahsan et al. [16] found that the majority of CT scans (93.1% and low predictive value of 0.74%, respectively) were normal or nondiagnostic, raising questions about its cost-effectiveness in routine ED evaluations, especially given the higher costs of CT compared to targeted clinical assessments alone. These findings echo broader critiques of CT overuse in dizziness, as highlighted by Kerber et al. [22], who noted that CT’s primary value lies in excluding hemorrhage or mass lesions rather than identifying ischemic etiologies. However, CT retains a pragmatic role in resource-limited settings or for rapid triage of high-risk patients, particularly when MRI is unavailable or contraindicated [23].

A critical insight from this review is the importance of clinical predictors in guiding imaging selection. Older age, male sex, cardiovascular risk factors, and the presence of neurologic signs were consistently associated with higher yields for MRI [12,14]. These predictors align with validated clinical decision tools, such as the STANDING algorithm [24], which stratifies dizziness patients based on stroke risk. For example, Hammoud et al. [14] found that MRI detected infarcts in 11.5% of patients with vague symptoms and negative CT, with positive findings strongly correlated with hypertension, diabetes, and prior stroke. This suggests that selective MRI use in high-risk populations could optimize diagnostic efficiency while mitigating unnecessary costs-a point emphasized by Ahsan et al. [16], who cautioned against routine MRI for low-risk patients due to its high expense and marginal incremental benefit.

The therapeutic impact of advanced imaging also warrants discussion. While MRI’s diagnostic superiority is clear, its influence on clinical management appears modest in some contexts. Fakhran et al. [15] reported that only 1-2% of findings from CT angiography or contrast-enhanced MRI led to management changes, underscoring a disconnect between detection and actionable outcomes. This mirrors broader debates about the “diagnostic-therapeutic gap” in neuroimaging, where increased sensitivity does not always translate to improved patient outcomes [25]. Nevertheless, MRI’s ability to identify subtle infarcts or alternative pathologies (e.g., vestibular neuritis and demyelination) can inform prognosis and secondary prevention, particularly in cases where CT is nondiagnostic [26].

The risk of bias assessment revealed methodological heterogeneity, with higher bias in retrospective studies lacking blinded reference standards [15,16]. In contrast, prospective designs like Hwang et al. [17] and Lozano et al. [19] provided robust evidence supporting MRI’s advantages. These variations highlight the need for standardized imaging protocols and prospective studies to minimize verification bias-a challenge noted in prior reviews [27].

Limitations

This review has several limitations. First, the predominance of retrospective studies introduces potential selection and verification biases, particularly in studies relying on clinical follow-up rather than standardized adjudication. Second, heterogeneity in imaging protocols (e.g., slice thickness and DWI sequences) may affect generalizability, as seen in Lozano et al.’s [19] findings on thin axial versus coronal DWI. Third, the exclusion of non-English studies and those behind paywalls may have omitted relevant data. Finally, the focus on acute ED settings limits applicability to outpatient or chronic dizziness evaluations.

## Conclusions

MRI outperforms CT for diagnosing acute dizziness in emergency settings, particularly for detecting posterior fossa infarctions and subtle ischemic strokes, with diagnostic yields of 12-27% versus CT’s 0.7-15%. While CT remains useful for rapid hemorrhage exclusion, MRI’s superior sensitivity - especially with thin-slice DWI protocols - makes it the preferred modality when stroke is suspected. However, its use should be guided by clinical risk stratification, incorporating age, vascular risk factors, and neurological signs to optimize resource utilization. These findings support developing evidence-based imaging pathways that prioritize MRI for high-risk patients while avoiding unnecessary CT in low-risk cases. Ultimately, this approach aims to reduce missed diagnoses without overutilizing advanced imaging.

## Appendices

**Table 4:** Search strategy for each database

Database	Search strategy
PubMed	("Dizziness"[Mesh] OR dizziness OR vertigo OR lightheadedness) AND ("Magnetic Resonance Imaging"[Mesh] OR MRI OR "magnetic resonance imaging") AND ("Computed Tomography"[Mesh] OR CT OR "computed tomography") AND ("Emergency Service, Hospital"[Mesh] OR "Emergency Department" OR "Emergency Room" OR ED) AND (acute OR sudden OR abrupt)
British Medical Journals (BMJ database)	(dizziness OR vertigo OR lightheadedness) AND (MRI OR "magnetic resonance imaging") AND (CT OR "computed tomography") AND ("Emergency Department" OR "Emergency Room" OR ED) AND (acute OR sudden OR abrupt)
Scopus	TITLE-ABS-KEY (dizziness OR vertigo OR lightheadedness) AND TITLE-ABS-KEY (MRI OR "magnetic resonance imaging") AND TITLE-ABS-KEY (CT OR "computed tomography") AND TITLE-ABS-KEY ("Emergency Department" OR "Emergency Room" OR ED) AND TITLE-ABS-KEY (acute OR sudden OR abrupt)
IEEE Xplore	("All Metadata": dizziness OR vertigo OR lightheadedness) AND ("All Metadata": MRI OR "magnetic resonance imaging") AND ("All Metadata": CT OR "computed tomography") AND ("All Metadata": "Emergency Department" OR "Emergency Room" OR ED) AND ("All Metadata": acute OR sudden OR abrupt)
Web of Science	TS=(dizziness OR vertigo OR lightheadedness) AND TS=(MRI OR "magnetic resonance imaging") AND TS=(CT OR "computed tomography") AND TS=("Emergency Department" OR "Emergency Room" OR ED) AND TS=(acute OR sudden OR abrupt)
